# Is the Activation of the Postsynaptic Ligand Gated Glycine- or GABA_A_ Receptors Essential for the Receptor Clustering at Inhibitory Synapses?

**DOI:** 10.3390/biomedicines13081905

**Published:** 2025-08-05

**Authors:** Eva Kiss, Joachim Kirsch, Jochen Kuhse, Stefan Kins

**Affiliations:** 1 Institute of Anatomy an and Cell Biology, University of Heidelberg, 69120 Heidelberg, Germany; eva.kiss@uni-heidelberg.de (E.K.);; 2 Department of Cellular and Molecular Biology, George Emil Palade University of Medicine, Pharmacy, Science and Technology of Târgu Mures, 540142 Târgu Mures, Romania; 3 Department of Human Biology and Human Genetics, University of Kaiserslautern, 67663 Kaiserslautern, Germany

**Keywords:** glycine receptors, GABAA receptors, receptor clustering, gephyrin

## Abstract

One major challenge in cellular neuroscience is to elucidate how the accurate alignment of presynaptic release sites with postsynaptic densely clustered ligand-gated ion channels at chemical synapses is achieved upon synapse assembly. The clustering of neurotransmitter receptors at postsynaptic sites is a key moment of synaptogenesis and determinant for effective synaptic transmission. The number of the ionotropic neurotransmitter receptors at these postsynaptic sites of both excitatory and inhibitory synapses is variable and is regulated by different mechanisms, thus allowing the modulation of synaptic strength, which is essential to tune neuronal network activity. Several well-regulated processes seem to be involved, including lateral diffusion within the plasma membrane and local anchoring as well as receptor endocytosis and recycling. The molecular mechanisms implicated are numerous and were reviewed recently in great detail. The role of pre-synaptically released neurotransmitters within the complex regulatory apparatus organizing the postsynaptic site underneath presynaptic terminals is not completely understood, even less for inhibitory synapses. In this mini review article, we focus on this aspect of synapse formation, summarizing and contrasting findings on the functional role of the neurotransmitters glycine and γ-aminobutyric acid (GABA) for initiation of postsynaptic receptor clustering and regulation of Cl^−^ channel receptor numbers at inhibitory synapses gathered over the last two decades.

## 1. Introduction

**Figure 1:**
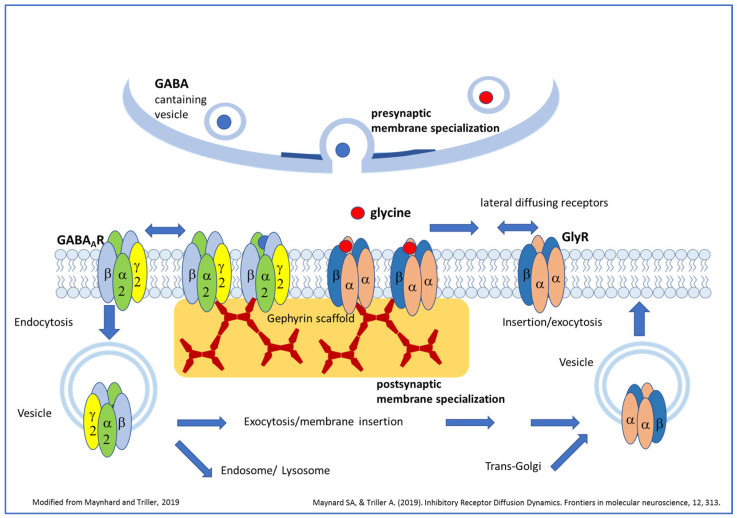
Overview of GABA_A_R and GlyR trafficking and putative roles of ligand binding for postsynaptic receptor clustering (modified from [16]). Vesicles carrying pentameric GlyRs or GABA_A_R are transported after segregation from the trans-Golgi compartment to the plasma membrane. After fusion of receptor-carrying vesicles with the plasma membrane, receptors diffuse in the plane of the membrane and might be trapped by the gephyrin scaffold beneath the postsynaptic membrane specialization. Here, several receptors can aggregate together, forming clusters. Receptors can also diffuse out from the postsynaptic membrane and might be removed from the cell surface by endocytosis. From this state they can be exocytosed again or might fuse with the early/late endosome for lysosomal degradation. Both neurotransmitters are able to induce the formation of new GlyR or GABA_A_R clusters; however, neurotransmitter binding seems to be essential only for GlyR cluster formation but not for the initial postsynaptic clustering of GABA_A_Rs.

One important goal of cellular neuroscience is to disclose the cellular and molecular mechanisms involved in regulating the assembly of chemical synapses, ensuring rapid synaptic transmission. Most synapses are established during development following genetically determined intrinsic mechanisms, but synapses continue to be formed and modulated in strength in the mature nervous system throughout life. Adjustments in the strength of neuronal connections occur in part postsynaptically by changes in the number of receptors at the postsynaptic sites [1,2,3]. The number of receptors that cluster postsynaptically depends first on lateral diffusion events from the extrasynaptic plasma membrane, where the membrane insertion of receptors carrying intracellular vesicle pools might occur (Figure 1). Receptor subunits are initially translated, folded, and assembled into complete receptor complexes within the rough endoplasmic reticulum (RER). Interestingly, in addition to the somatic region, a local de novo synthesis of γ-aminobutyric acid A-receptors (GABA_A_Rs) and even of gephyrin, a main scaffold protein at the inhibitory postsynapse, was also demonstrated in dendrites as driven by a reduced expression of two miRNAs and regulated by N-methyl-D-aspartate receptor (NMDAR) signaling [4,5]. The delivery of receptor-containing vesicles to the plasma membrane results from active cytoskeletal transport after segregation from the trans-Golgi compartment or previous endocytosis from the extrasynaptic plasma membrane. Indeed, a part of extrasynaptically localized membrane neurotransmitter receptors are internalized with the potential for recycling or lysosomal degradation [6,7]. Accordingly, a number of highly regulated processes implicating multiple intracellular signaling cascades are likely to affect receptor localization, stabilization, and maintenance at the synapse [8,9]. However, the specific mechanisms that target receptors from intracellular or extrasynaptic compartments and keep them at synapses are still not clear and even less well understood for inhibitory synapses compared to glutamatergic synapses. Cell adhesion molecules in cooperation with synaptic scaffold proteins were proposed to play a key role in facilitating the accumulation of surface neurotransmitter receptors at postsynaptic sites and modulating receptor dynamics and were reviewed recently in great detail [9,10]. What is the contribution of neurotransmitter signals to the organization of the appropriate neurotransmitter receptor clusters at the synapse? Searching for answers to this seemingly simple question, here we proposed to review studies that have provided insights into the part ligand activation itself accomplishes in regulating the formation and maintenance of postsynaptic glycine receptor (GlyR) and GABA_A_R clusters at inhibitory synapses.

The neurotransmitters glycine and GABA mediate fast inhibitory neurotransmission in the mature nervous system through activation of GlyRs and GABA_A_Rs, respectively, which both belong to the superfamily of heteropentameric ligand-gated ion channels [11]. Under normal physiological conditions, activation of GlyRs and GABA_A_Rs by ligand binding opens a chloride-permeable ion channel, allowing chloride ions (Cl^−^) to enter the neuron, resulting in membrane hyperpolarization and neuronal inhibition. However, in immature neurons, due to the relatively high intracellular Cl^−^ concentration, activation of GlyRs and GABA_A_Rs leads to chloride efflux and consequently to a depolarization of the plasma membrane. Therefore, GlyRs and GABA_A_Rs are mostly excitatory during development and, through the activation of voltage-gated Ca^2+^ channels, orchestrate numerous Ca^2+^ -dependent processes [12,13,14] that may underlie some type and developmental stage dependent differences in receptor clustering properties. Ca^2+^ as a second messenger influences the activation of kinases like Ca^2+^/calmodulin-dependent protein kinase type II (CaMKII) and phosphatases like calcineurin, which in turn, by modifications of receptors and other proteins, affect receptor trafficking and other intracellular signaling pathways that ultimately lead to changes in gene expression and protein synthesis. Altogether these will shape postsynaptic receptor cluster formation and contribute to inhibitory synaptic plasticity [8,15].

In this manuscript we review in a time-course manner publications and discuss knowledge converged over the last 25 years with special focus on the role glycine and GABA signals might have in both the immature state and mature nervous system on the two main aspects of postsynaptic receptor cluster formation: First, the initiation of the de novo formation of receptor clusters, thus related to the number of postsynaptic clusters in a given dendritic or somatic region, and second, the increase and decrease in cluster size as one structural correlate of synaptic plasticity. In this context, the clustering of gephyrin, thought to be the main postsynaptic scaffold and organizer of inhibitory synapses [17,18], will also be discussed.

## 2. Postsynaptic Clustering of the Strychnine-Sensitive GlyRs in Early Developmental Stages In Vitro

**Figure 2:**
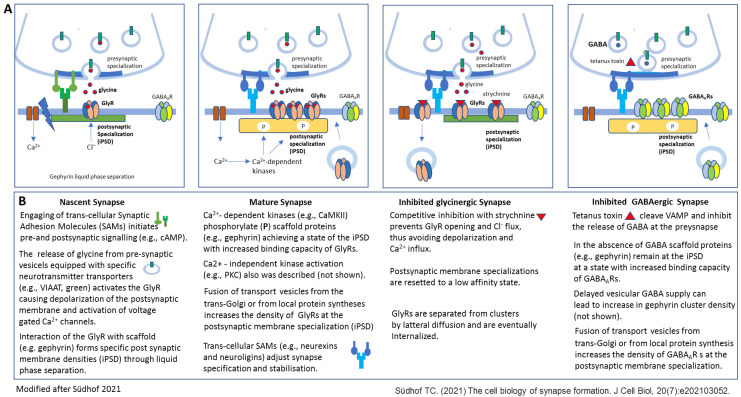
Different stages of inhibitory synapse formation and effects of experimental activity blockade with focus on the postsynaptic membrane specializations (modified after [8]). Synapses initiate as nascent contacts that mature into plastic synaptic connections. (**A**) Schematic view of a nascent glycinergic synapse (first), a mature glycinergic synapse (second), a glycinergic synapse (third), and GABAergic synapse after experimental activity blockade (fourth). In nascent synapses Synaptic Adhesion Molecules (SAMs) mediate initial transneuronal interactions. For example, latrophilins, specifically latrophilin-1 at inhibitory synapses, are thought to initiate intracellular signaling cascades that organize inhibitory synaptic specializations. Binding of the postsynaptic neuroligins (specifically NLG2 at inhibitory synapses) to its presynaptic partners, neurexins, is proposed to shape subsequent synapse maturation and properties (second). Inhibition of synapses with the high-affinity competitive antagonist strychnine results in altered GlyR clustering due to elimination of GlyRs from the postsynaptic membrane specialization (third). Inhibition of GABA release due to VAMP cleavage through tetanus toxin does not affect GABA_A_R clustering at the postsynaptic membrane specialization. (**B**) Short description of the events presented in A. Further details are provided in the manuscript text. NLG—neurologin 2, VAMP—vesicle-associated membrane protein.

GlyR cluster formation at postsynaptic membranes in relation to neurotransmitter-dependent receptor activation was first analyzed in 1998 by two independent studies [19,20]. Both studies used the same experimental system of activity blockade by strychnine of cultured rat spinal cord neurons, which recapitulate the developmental sequence of GlyR expression. Strychnine, an alkaloid extracted from the Indian tree *Strychnox nux vomica,* is a potent competitive GlyR antagonist that blocks the binding of glycine to its Cl^−^ channel receptor, particularly within the spinal cord. Electron cryo-microscopy studies have provided insights into how strychnine binding to the GlyR promotes receptor domain rearrangement leading to rotation of the transmembrane domain toward the pore axis, occluding the ion conduction route [21,22]. The high affinity and specificity of strychnine for GlyRs make strychnine an ideal antagonist ligand to study the dynamics of GlyR topography in response to activity in cell culture or in vivo conditions. The model primarily targets the α1 GlyRs; thus, the findings might not accurately reflect the roles of other α GlyR subtypes with more restricted expression patterns. Furthermore, prolonged strychnine exposure can lead to changes in GlyR mRNA and protein levels, affecting the overall expression levels of GlyRs, while at higher concentrations its activity extends beyond GlyRs, all representing limitations that have to be considered when interpreting results [23,24]. In the work of Levi et al., chronic inhibition of the GlyR with increasing strychnine concentrations for 7 or 11 days reduced in a group of strychnine-sensitive neurons the number of synaptic GlyR clusters, visualized as mAb4a (known to recognize all GlyR α subunits) immunoreactivity, in a dose-dependent manner. In addition, the size of the few GlyR clusters found on the surface of strychnine-responsive neurons was also significantly smaller than those seen on the membrane of untreated neurons, suggesting that GlyR clusters were first destabilized [19]. The second study reported similar results in the sense that strychnine treatment for 8 days resulted in a significantly lower number of mAb4a co-localizations with presynaptic terminals, indicating postsynaptic GlyR clusters [20] (Figure 2). Interestingly, in this study, a very similar effect could be measured upon inhibition of voltage-dependent Ca^2+^ channels (verapamil and nifedipine), suggesting that the depolarizing effect of the GlyR activation in immature neurons causing the opening of L-type Ca^2+^ channels with subsequent Ca^2+^ influx is needed for GlyR clustering at developing postsynaptic sites. The downstream signaling mechanisms leading to the receptor cluster formation were not further characterized.

In both studies, in the subgroup of strychnine-responsive neurons, the reduced number of post-synaptic GlyR clusters was correlated with the intracytoplasmic accumulation of larger GlyR-containing structures next to the nucleus, which were not colocalized with either cis-Golgi apparatus or RER, arguing against neosynthesized GlyRs [19]. Based on these observations, the authors hypothesized that strychnine-mediated blocking of postsynaptic GlyRs ultimately led to receptor removal from the dendritic and somatic membrane surface via endocytosis, which was not compensated by new GlyR incorporation and lateral diffusion into the postsynaptic receptor cluster [19,20]. However, this hypothesis was refuted a few years later by the same group from the Triller lab. Evaluating GlyR clustering upon short-term exposure of neurons to strychnine (5 h, 1 h), they observed that the perinuclear accumulation of immunoreactive GlyRs precedes the disappearance of the receptors from postsynaptic sites [25]. By labeling the lumen or membrane of the endocytic compartments, they could show that the strychnine-induced redistribution and accumulation of GlyR is not the result of the endocytosis of plasma membrane receptor molecules but rather affects the activity blockade by strychnine, the receptors postsynaptic anchoring, and/or the reloading of postsynaptic clusters due to altered protein exocytic trafficking [25]. Anyway, these studies assign transmitter-induced activity a pivotal role in GlyR clustering at early stages of development but less for the maintenance of differentiated postsynaptic sites since when strychnine was added after 8 Div, when GlyR clusters had already formed, the postsynaptic localization of GlyR remained the same for several days [20]. Furthermore, it has to be emphasized that these conclusions were restricted to a subpopulation of strychnine-responsive neurons within the spinal cord culture containing also neurons with many GlyR clusters even upon strychnine treatment. Besides, it has to be noticed that, in addition to the above-mentioned limitations of the chronic strychnine treatment model, the presence or absence of glycine in the cell culture medium or altered conditions of the energy metabolism might have also influenced the results of these experiments.

## 3. Postsynaptic Clustering of GlyRs in Early Developmental Stages In Vivo

More than 10 years after the above-described in vitro studies investigated Yamanaka et al., whether neurotransmitter signaling is required for the initial assembly of GlyRs throughout synapse formation in vivo, using chronic strychnine treatment during the early development of zebrafish [26]. Bath-applied strychnine to zebrafish embryos from 22 hpf (hours postfertilization), before GlyR clusters had formed, hindered almost completely the formation of postsynaptic GlyR clusters at Mauthner-cells (M-cells) surface, which are a single pair of reticulospinal neurons in the hindbrain, receiving many inhibitory inputs from glycinergic interneurons on their somatic and dendritic membrane. Importantly, and in concordance with the earlier in vitro observations [19,20], after strychnine removal, GlyR clusters appeared on the surface of M-cells and even increased in density over time. Furthermore, labeling GlyR clusters with mAb4a antibody after strychnine application for 24 h at later stages of zebrafish development, when GlyR clusters had already been formed, the previously assembled GlyR clusters disappeared from the surface of M-cells in the early larval stages (2–3 dpf) and were markedly diminished in late larvae and juveniles (10–11 dpf, 15–16 dpf, 30–31 dpf) (Figure 2). The authors proposed that the age-dependent decrease in the activity-dependent maintenance of GlyR clusters might be at least partly assigned to the reduction of intracellular Cl^−^ concentration along neuron development, although this is questionable since in immature spinal cord neurons in vitro no effect of late strychnine application on receptor cluster maintenance was observed [20]. However, this study was the first and so far the only one demonstrating, by immunolabeling supported by electrophysiological recordings, that glycinergic transmission is indeed necessary for GlyR cluster formation in vivo and also for maintenance of previously formed GlyR clusters, at least until juvenile periods. Mechanistically this work confirmed the functional role of the GlyR activation in vivo causing depolarization and opening of L-type Ca^2+^ channels since the specific inhibition of these channels as well as of the calcium-/calmodulin-dependent protein kinase II (CaMKII) resulted in the same robust reduction of GlyR clusters as the strychnine treatment itself [26]. The very fast response to strychnine application, namely, eliminating GlyR clusters within one hour, strongly indicated as a cellular mechanism for the disappearance of postsynaptic GlyRs after strychnine treatment the increased lateral diffusion of receptors out of the postsynaptic membrane area due probably to altered anchoring at the gephyrin scaffold, known to be modulated by CaMKII-dependent phosphorylation [27]. CaMKIIα, the predominant CaMKII isoform in the hippocampus and neocortex, is activated by Ca^2+^ influx and subsequent calmodulin binding, leading to its autophosphorylation and sustained activity [15]. Activated CaMKII likely promotes gephyrin phosphorylation, which accelerates the GlyR cluster formation at the postsynaptic sites. Indeed, in a later study, it could be shown that the phosphorylation of gephyrin at Ser325 by CaMKIIα promotes the synaptic accumulation of GlyR on zebrafish M-cells through enhancement of the gephyrin-GlyR binding, increasing inhibitory glycinergic inputs [28].

Whether the long-term strychnine treatment effects might also involve alterations in the expression and/or intracellular transport of GlyRs was not clarified within this study [26].

## 4. Postsynaptic GlyR Clustering in Mature Neurons In Vitro

Similar conclusions were drawn by a study published four years later [29] analyzing the consequences of chronic GlyR inhibition on postsynaptic glycine receptor clustering in mature-state spinal cord neuron cultures (Div13–15), in which, as is well known, the receptor activation is more likely to cause hyperpolarization of the postsynaptic membrane than depolarization, as in the immature state. In this study, the chronic inhibition by strychnine for 14 days resulted in a decrease in the size of the postsynaptic GlyR clusters detected as GlyRα1 puncta apposed to vesicular inhibitory amino acid transporter (VIAAT) signals. Applying a combination of fluorescence recovery after photobleaching (FRAP) and fluorescence loss in photobleaching (FLIP) techniques, it was convincingly presented that the smaller clusters emanated rather from the increased diffusive property of the receptors in the plane of the membrane after inhibition than from a decline in exocytosis of new intracellular GlyRs into the plasma membrane. Furthermore, the phenomenon could be reversed since wash-out of strychnine and intrinsic activation of GlyRs by glycine for one hour led to enlargement of the synaptic GlyR clusters coincident with a decrease in receptors lateral diffusion at synaptic sites. Thus, these results evidenced the important role of glycinergic transmission for the formation of synaptic GlyR clusters also in mature state neurons. However, the experiments testing the effect of strychnine blocking on previously activated GlyRs in Div14 neurons indicated that GlyR activation is not essential for their maintenance at the synapse. Importantly, the alteration in cluster size was not dependent on CaMKII activity but was shown to be regulated by protein kinase C (PKC) in a Ca^2+^-independent manner. Cytosolic PKC activated by diacylglycerol or other lipid- or non-lipid molecules translocates to the plasma membrane [30,31], where it phosphorylates specific serine residues of the GlyR, changing channel properties and receptor trafficking as well as interactions between GlyRs and proteins like gephyrin [32]. In this context, it is of relevance that PKC phosphorylation at the Ser403 site was found to reduce the binding affinity between the GlyR and gephyrin, resulting in acceleration of the receptor’s diffusion in the plasma membrane and reduced GlyRs accumulation at synapses [33].

Further, it is worth mentioning that the in vitro strychnine inhibition experiment with mature state spinal cord neurons also revealed additional differences in comparison to the earlier findings in young spinal cord neurons. First of all, the strychnine treatment did not alter the number of either total or synaptic GlyR clusters in mature cultures. Additionally, site-specific application of glycine successfully induced the formation of new GlyR clusters in the mature-state spinal cord neurons, an experimental result that was reported in one of the first GlyR cluster studies from 1998 to be not effective in immature neurons [20].

In conclusion, a limited number of publications have shown until now that transmitter-dependent GlyR activation affects GlyR clustering both in the early membrane depolarization and later hyperpolarization states by regulating the initial formation and maintenance of GlyR clusters at the postsynaptic membrane site or GlyR receptor cluster size, respectively. Mechanistically, the activation might imply different kinds of phosphorylations through either CaMKII in early or PKC in later developmental stages, resulting in modulation of the lateral mobility of GlyRs, i.e., persistence of the GlyRs at the postsynaptic membrane specialization defined probably through the submembranous scaffold of gephyrin. Whether or not GlyR cluster formation can occur without any glycine binding is not known.

## 5. Gephyrin Cluster Formation at Glycinergic Synapses

In contrast to the obvious dependence of GlyR cluster formation and stability at postsynaptic membrane sites on glycinergic transmission described above, for the scaffold protein gephyrin a different scenario emerges. At postsynaptic sites, all GlyRs and the majority of GABA_A_Rs connect to gephyrin, which is thought to be the major scaffold protein of the postsynaptic density at inhibitory synapses, although meanwhile numerous additional proteins were identified to contribute to the proteome of inhibitory synapses [34,35]. Gephyrin has been demonstrated to regulate diffusion properties of GlyR and GABA_A_R receptors, thus influencing the size and density of postsynaptic receptor clusters [17,18]. The mechanisms underlying gephyrin-receptor interactions leading to receptor clustering are relatively well defined; in particular, the binding domains of both gephyrin and the intracellular linker region of the GlyRβ subunit could be determined through crystallography and in vitro binding assays at atomic resolution level [36].

However, how these interactions and the subcellular distribution of gephyrin are affected by receptor activation is not well understood. Structurally, gephyrin consists of an N-terminal trimer forming the G-domain, a C-terminal dimer forming the E-domain, and a connecting linker region. A number of studies clearly disclosed that these domains interact to create a hexagonal lattice that can anchor inhibitory receptors at postsynaptic sites and interact with various synaptic and cytoskeletal proteins (reviewed in 18). Later studies evidenced that this lattice can undergo phase separation, leading to the formation of condensed sheet-like structures termed inhibitory postsynaptic density (iPSD), particularly when gephyrin is bound to GlyRs or GABA_A_Rs, which can be further fine-tuned by different gephyrin-binding proteins such as neuroligin 2 (NLG2) [37,38]. Since the specific interaction of the dimeric E-domain of gephyrin with GlyRs or GABA_A_R multimers seems to be required for the iPSD formation, linker region phosphorylation at different residues or binding of other synaptic proteins regulates gephyrin-mediated GlyR and GABA_A_R clustering [37]. Phosphorylation of specific serine and threonine residues on gephyrin involving various kinases such as Glycogen Synthase Kinase 3 beta (GSK3β), Cyclin-dependent kinase 5 (CDK5), protein kinase A (PKA), and CaMKII can modulate its interaction with both receptors and other proteins involved in synapse formation and plasticity [28,39,40,41,42].

The postsynaptic accumulation of gephyrin, in comparison to GlyR clusters, was observed also upon chronic strychnine treatment in both immature (Div 7) [19] and mature spinal cord neurons (Div 14) [28]. In these studies, neither chronic strychnine application nor the washout of chronic strychnine with subsequent GlyR activation modified the subcellular distribution and the number of gephyrin clusters detected by the mAb7 antibody. In contrast, the study of Kirsch et al., reported that similarly to the GlyR clusters, gephyrin, revealed by the same monoclonal antibody 7a, “was no longer detected at postsynaptic sites of DIV8 spinal cord neurons after chronic strychnine treatment but was seen instead at sites of focal cell attachment”. Consistently, Ca^2+^-channel blockers affected the gephyrin distribution in the same way [20]. How these different observations might be explained is not totally clear. At a later time point it was shown that the binding of the monoclonal antibody mAb7a to gephyrin is dependent on the phosphorylation of gephyrin at Ser270 [42]. Since the same developmental stage spinal cord neurons were used in both of these studies, some differences in the cell culture system conditions (e.g., cell density, medium composition) might have resulted in different phosphorylation states of gephyrin at this site, causing discrepancies in the detection pattern of gephyrin clusters by the mAb7a antibody. Additionally, it is known that even within the same culture, different neurons can have varying levels of differentiation and maturity, leading to inconsistencies between experiments. Nevertheless, the latter data are in some way supported by the study of Maas, C et al., showing in hippocampal neurons that inhibition of the GlyRs reduced the anterograde transport of gephyrin-GlyR complexes on microtubule tracks into the dendrites, thus resulting in significantly decreased gephyrin signal number and fluorescence intensity in neurites and in a net accumulation of newly synthesized gephyrin in the cytoplasmic compartment [43]. The alteration in microtubule-dependent transport activity seen with the strychnine-mediated GlyR blockade could be mimicked by excitatory (AMPA receptor) activity. This suggests that the observed transport deficits were also in these cases probably indirectly caused by the GlyR activation, namely due to the depolarization of the postsynaptic membrane inducing Ca^2+^ influx by voltage-dependent Ca^2+^ channels characteristic of young stages of spinal cord and also hippocampal neurons. Unfortunately, in this study the subcellular distribution of GlyRs was not analyzed; thus, it remains unclear whether the large intracellular GlyR accumulations seen in the studies from the Kirsch and Triller laboratories [19,20] would have been confirmed, which would be of interest, as in the former studies with strychnine inhibition conditions, on the other hand, no large intracellular gephyrin clusters were observed.

In conclusion, a handful of studies analyzing the accumulation of gephyrin and its effect on GlyR clustering at the postsynaptic sites in correlation with glycine-dependent synaptic activity reported contrasting results in regard to gephyrin clustering. Further studies have to clarify whether or not and when and how glycine-dependent neurotransmission might affect gephyrin aggregation supporting GlyR clustering at glycinergic synapses. Anyhow, the findings of these studies indicate that synaptic activity could alter the distribution of GlyR receptors and eventually other postsynaptic signaling molecules without affecting that of gephyrin, suggesting that distinct regulatory mechanisms might be involved in controlling the behavior of the different postsynaptic site components.

## 6. Postsynaptic Clustering of the GABA_A_ Receptors In Vitro and In Vivo

Two years after the two crucial studies showing that in cultured spinal cord neurons GlyR activation is needed for the formation and stabilization of postsynaptic GlyR clusters, a study published in the year 2000 using cultures of mature rat hippocampal neurons (21–28 Div) reported that GABA input is not necessary for clustering the GABA_A_Rs in pyramidal cells [44]. More exactly, the study revealed that isolated hippocampal pyramidal cells in microisland cultures thus deprived of GABA input formed GABA_A_R clusters, evidenced in this study by immunostaining for the β2/3 subunits of the GABA_A_R. Even more, the authors indicated that in the absence of input from GABAergic axons, these receptor clusters were localized opposite to glutamatergic presynaptic terminals, strongly indicating that the neurotransmitter itself does not represent the specificity-conferring signal for GABA_A_R clustering at the postsynaptic site. Shortly after, this statement was supported by two other studies in in vivo experimental conditions. In the first in vivo study, the analysis of Munc18-1 deficient mice revealed that even in the case of a complete loss of neurotransmitter secretion during development, including both evoked and spontaneous vesicular release, functional GABA_A_R clusters at postsynaptic sites are formed in the brain of these mice [45]. Munc18-1 is a presynaptic protein with multiple activities during soluble N-ethylmaleimide-sensitive factor attachment protein receptor (SNARE)-regulated exocytosis, including docking the neurotransmitter-transporting presynaptic vesicle, thus essential for membrane fusion and neurotransmitter release. Loss of Munc18-1 causes severe defects in neurotransmitter release [46]. GABA iontophoresis in Munc-18 mutant brain slices caused a normal postsynaptic response. The protein composition of the GABAergic postsynaptic sites was not addressed in this study. However, the severe neurodegeneration observed after the initial successful assembly of synaptic connectivity suggested that since the synapse formation might not require neurotransmitter secretion, it might be important for the maintenance of already established synaptic connections. In the second in vivo study three years later, the authors analyzed the formation of GABAergic neuromuscular junctions in *Caenorhabditis elegans* with the unc-25 mutation lacking any GABA synthesis [47]. Unc genes were originally identified in the nematode worm *Caenorhabditis elegans* and are a group of genes that, when mutated, result in uncoordinated (hence “UNC”) movement. Specifically, the unc-25 gene encodes glutamic acid decarboxylase (GAD), the biosynthetic enzyme for GABA [48]. By means of immunohistochemistry and GFP-tagged proteins, it turned out that unc-25 mutants formed GABA_A_R clusters at postsynaptic sites in the absence of GABA, and these clusters were indistinguishable from those of wild-type animals, including cluster size and synapse densities all along the time course evaluated. Shortly afterwards, the work of Harms and Craig [49] and then a more recent study [50] evidenced, just in line with the in vivo findings, that despite early block of any neurotransmitter release, GABAergic inhibitory synapses are also established in cultured hippocampal neurons (16–18 Div). In these experiments tetanus toxin was used to chronically cleave vesicle-associated membrane protein 2 (VAMP2), a central protein for SNARE complex assembly, vesicle fusion, and transmitter release. Tetanus toxin is a metalloprotease that specifically targets VAMP (also known as synaptobrevin), an essential component of the neurotransmitter release machinery. It inhibits particularly the release of GABA and glycine at inhibitory synapses, leading to increased excitability and development of spontaneous seizures, making the tetanus toxin model a useful tool for studying epilepsy [51,52]. This model also allows us to investigate the mechanisms of synaptic transmission, including the impacts of disrupted neurotransmitter release both in vivo and in vitro. However, it is important to be aware of the model’s limitations, including concentration-dependent effects, retrograde transport, and potential for off-target effects [53,54]. Immunolabeling for the GABA_A_R α2 and γ2 subunits evidenced receptor clustering opposite to GAD-positive terminals in tetanus toxin-treated as in control neurons. Neither GABA_A_R distribution nor size of GABA_A_R clusters was affected by inactivity [49] (Figure 2); however, when three-dimensional, structured illumination microscopy (3D-SIM) was used to analyze the nanoscale architecture of the inhibitory postsynaptic regions, the summed GABA_A_R (γ2) volume at the subsynaptic domains per synapse was significantly decreased at chronically silenced synapses, probably due to discrete decreases in both the number/synapse and individual volume of GABA_A_R subsynaptic domains [55]. This could be of importance since in the last years super-resolution microscopy methods like 3D-SIM and dSTORM could evidence the nanoarchitecture of synapses in which neurotransmitter receptors, scaffold proteins, and adhesion molecules cluster into nanoscale subsynaptic domains (SSD) [56,57]. Recent cryo-electron tomography (cryo-ET) studies could confirm the precise nanoscale organization of proteins in SSDs [58] that seems to be crucial for efficient and regulated synaptic transmission. Changes in the nanoscale organization of synapses, including GABA_A_Rs, such as those induced by activity, might enable dynamic regulation of synaptic function [57,59].

Although in the experimental models established to inhibit presynaptic neurotransmitter secretion and used in the above-mentioned studies, a release of residual GABA or other factors that might activate GABA_A_ receptors cannot be totally excluded, altogether these studies disclaim the requirement of local GABA_A_ receptor activation for the postsynaptic clustering of GABA_A_ receptors, as was the case for glycine receptors. This assigns a different role for the ligand binding with respect to the assembly of postsynaptic sites at GABAergic than at glycinergic synapses. In this context, of relevance is the information provided by the study of Levi et al., comprising a comparative analysis of receptor movement by single-molecule tracking technique in cultured spinal cord neurons (Div 10–13) combined with functional electrophysiological analysis and showing that GlyR but not GABA_A_R movement in and out of the postsynaptic membrane specialization is regulated by neuronal activity [60]. The main finding of this study was that GlyRs and GABA_A_Rs differ in the regulation of their diffusion properties by activity, the activity-dependent modulation of GlyR diffusion properties leading to changes in GlyR numbers at synapses, but without affecting gephyrin cluster immunoreactivities. Moreover, this study also demonstrated a specific NMDAR-dependent calcium-mediated regulation of GlyRs but not of GABA_A_Rs lateral diffusion, synaptic density, and mIPSC amplitude. This might represent a possible rapid homeostatic regulation mechanism at the inhibitory glycinergic or mixed glycine-GABAergic synapses to increased excitation [60]. In addition, specific phosphorylation of GABA_A_R subunits was indicated as another molecular mechanism that can govern receptors synaptic targeting, including binding to gephyrin [14,61]. Namely, cAMP-dependent PKA-mediated phosphorylation of the α2 subunit at Ser359 was identified to regulate scaffold binding and GABA_A_R cluster density at inhibitory synapses, thereby regulating the strength of inhibition in rat primary and cortical cell cultures and mouse brain slices [62].

## 7. Gephyrin Clustering at GABAergic Synapses

Unfortunately, only a few studies analyzed both GABA_A_R and gephyrin clustering under similar conditions of altered neurotransmitter-dependent synaptic activity. The early study of Rao et al. reported that in the absence of GABA input, not only GABA_A_R clusters but also gephyrin clusters are formed [44]. In isolated mature hippocampal pyramidal neurons, both GABA_A_R and gephyrin clusters were found opposed to presynaptic synaptophysin puncta in the absence of GABAergic innervation, although a detailed qualitative and quantitative analysis for gephyrin clusters was not carried out in this study. In tetanus toxin-treated hippocampal neurons, gephyrin clusters were also successfully recruited at postsynaptic sites, where they co-clustered with α2 and γ2 GABA_A_R subunits. No changes in gephyrin cluster density were noticed in the absence of GABA release [49]. However, in a later study when under the same experimental conditions of chronic tetanus toxin inhibiting hippocampal neurons (Div16), the postsynaptic architecture of inhibitory synapses was analyzed by super-resolution microscope (SIM). By unchanged number, the average size of gephyrin subsynaptic domains was markedly reduced, in contrast to the discrete changes of GABA_A_R subsynaptic domains, and resulted in significantly smaller postsynaptic gephyrin volume [55]. Altogether, these results suggested that neurotransmission might not be essential neither for GABA_A_R nor for gephyrin cluster formation at GABAergic synapses, but it might play a role in recruiting further gephyrin molecules to already formed postsynaptic sites and the organization of gephyrin SSDs.

This view is, however, challenged by a study analyzing the formation of GABAergic postsynaptic assemblies in organotypic slice cultures of mouse cortex and in vivo by local release of GABA near dendritic branches by two-photon photolysis under conditions of high-frequency uncaging (HFU) during development [63]. Under both experimental conditions new gephyrin clusters were induced in distal dendritic areas of cortical neurons. The success rate of gephyrin cluster formation was in the range of about 55% in younger slices (6–8 Div) and was reduced in older stages (14–18 Div). Gephyrin clustering was not detected after glutamate HFU, but further pharmacological experiments with this system confirmed the dependence of the gephyrin cluster formation on Ca^2+^ influx through voltage-dependent L- and T-type Ca^2+^ channels, leading to the conclusion that the early depolarizing action of GABA can promote local synaptogenesis during cortical development. Unfortunately, GABA_A_R structural changes were not analyzed by immunofluorescence microscopy in this study, but functional analysis by measuring uncaging-evoked inhibitory postsynaptic currents (uIPSCs) from newly formed gephyrin puncta supported the presence of functional GABA_A_R clusters at these sites. Tetanus toxin-induced prevention of GABA release resulted in this study in reduced density of gephyrin puncta in distal apical dendrites of young cortical layer 2/3 pyramidal neurons and decreased the frequency of both miniature IPSCs (mIPSCs) and miniature EPSCs (mEPSCs) in slices and in vivo. Thus, these results support the view that early GABAergic inputs from cortical interneurons are essential for inhibitory (and even excitatory) synaptic connections during cortical development. A quite different experimental system was used in a later study, namely stem cell-derived human neurons as well as in vivo mouse neurons of purely glutamatergic identity, which were reprogrammed for ectopic GABA synthesis and release from presynaptic terminals [64]. This led to accumulation and activation of postsynaptic GABA_A_Rs, generating fully functional GABAergic synapses in adjacent neurons. In more detail, the formation of both gephyrin and GABA_A_R (alpha3) clusters was newly induced both at the dendritic segments as well as perisomatic regions of glutamatergic neurons without affecting cluster size, thus supporting that presynaptic GABA release itself can enable the organization of the corresponding postsynaptic apparatus. Noteworthy, at the end of the discussion part of the manuscript, the authors claimed that important levels of both gephyrin and GABA_A_R clusters were already present even in neurons that were not exposed to presynaptic GABA release. In this context, although neither the number nor the size of these preexisting clusters was apparently evaluated within this work, the findings of another study published more than 20 years before the latter manuscript are worth considering [65]. The authors of this manuscript communicated that in low-density hippocampal cultures (Div19) in the absence of GABA release at the surface of dendrites and soma of pyramidal cells, small (0.36 +/− 0.01 micrometer diameter) GABA_A_R clusters with heterogenous subunit composition were monitored. Upon GABAergic innervation the same cells formed 3–4-fold larger GABA_A_R clusters with higher receptor density and more defined subunit composition. Large clusters of GABA_A_Rs are opposed to GABAergic boutons and colocalized with large gephyrin clusters, whereas small clusters of GABA_A_Rs are colocalized with small clusters of gephyrin. Since the lateral diffusion of such large aggregates in the membrane is unlikely, the authors proposed that the local signals generated at GABAergic synapses induced the assembly of large synaptic GABA_A_R clusters and the parallel disappearance of the small GABA_A_R clusters around the synaptic area.

Thus, in the absence of GABAergic innervation, other signals, e.g., those generated at glutamatergic synapses, might induce the formation of small gephyrin and GABA_A_R clusters that can be associated also with other than GABAergic presynaptic contacts or might even have no presynaptic partners. In this line a basic level of preexisting GABA_A_ receptor clusters at the neuron’s surface is very likely to be present even in the absence of GABA; however, an instructive role of GABA to induce real newly formed GABAergic synaptic clusters is strongly supported by these studies. Interestingly enough, two years after informing about the signaling role of the neurotransmitter itself for postsynaptic accumulation of gephyrin and GABA_A_Rs, the same lab brought out a publication reporting that gephyrin promotes autonomous assembly and synaptic localization of GABAergic postsynaptic components without presynaptic GABA release [66]. In this study, the authors analyzed the cellular distribution of several GABAergic postsynaptic proteins in purely glutamatergic human neuronal cultures derived from induced pluripotent stem cells (iPS), showing that various GABA_A_R subunits, gephyrin, and even cell-adhesion molecules can coaggregate also at GABA-deficient subsynaptic domains. However, delayed vesicular GABA supply led to a significant increase in gephyrin cluster density without affecting cluster size, supporting that gephyrin–GABA_A_R clusters, developed in the absence of GABA transmission, can be further modulated by post-hoc GABA release.

Taken together, this latest study somehow confirmed the first set of publications that intrinsic molecular mechanisms promote gephyrin and GABA_A_R assembly to form postsynaptic clusters well suited to establish functional inhibitory synapses. In addition, similar to the instructive function of glycine to induce the formation of GlyR clusters, GABA also has the capability to promote the formation of GABA_A_R clusters and contribute to their maintenance at the postsynapse, probably by modulating gephyrin interactions with its multiple binding partners [64,66]. Several studies have shown that phosphorylation of gephyrin depending on the kinase and the phosphorylation site can either increase or reduce GABA_A_R clustering [42,67], but how these molecular mechanisms relate to neurotransmitter-induced synaptic activity was in general not studied. Activity-dependent GABA_A_R synapse remodeling was demonstrated by gephyrin phosphorylation involving CAMKII [27]. How ligand binding affects the binding of gephyrin to its other partner proteins, such as adhesion molecules, collybistin, a brain-specific guanine nucleotide exchange factor, and cytoskeletal proteins [17,18,68], or how activity might directly modulate these gephyrin binding partners to control postsynaptic plasticity is even less studied. Gephyrin’s direct or indirect interactions with adhesion molecules that mediate initial contacts between pre- and post-synaptic membranes (like latrophilins, neuroligins, IgSF9b, and N-cadherin) are thought to be important for receptor localization stabilizing the nascent synaptic structure [68] (Figure 2). Specifically, NLG2 has been shown to be localized at GABAergic inhibitory post-synapses and interacts with presynaptic neurexins to form a trans-synaptic complex [69]. Moreover, NLG2 stabilizes the open/active conformation of collybistin, allowing increased gephyrin clustering [70]. Importantly, NLG2 overexpression caused an increase in synapse number (synapsin stain), and elevation of iPSC amplitudes in hippocampal neurons that were reversed after chronic inhibition of neuronal network activities, indicating that NLG2 might mediate activity-dependent specification and validation of inhibitory synapses [71]. However, phosphorylation of NLG2 by PKA disrupted its interaction with the scaffold protein and decreased GABA_A_R abundance [72]. Even more, super-resolution microscopy studies have shown that NLGN2 forms SSDs that significantly overlap with gephyrin SSDs at the nanoscale level [73], and that acute cleavage of the extracellular domain of NLGN2 resulted in dispersion of GABA_A_Rs, leading to impaired synaptic transmission [59]. Interestingly, recent studies provided evidence for several transmembrane proteins such as LHFPL4/GARLH, Clptm1, and Shisa-7 that interact with GABA_A_Rs, modulating their trafficking and function [74], altogether further underscoring the complexity of inhibitory synapse biology.

## 8. Limitations and Perspectives

It is important to realize that in addition to the very different ways to approach the topic of neurotransmitter-dependent clustering of GlyRs or GABA_A_Rs at the postsynaptic sites, all these experiments were conducted in somewhat restricted conditions, mostly missing the entire complex environment where synapse formation normally happens, and therefore not all whole-context-dependent mechanisms might be sufficiently recognized. Especially, the in vitro studies might lack the effects of the microvasculature as well as complex cellular interactions and neuronal networks found in the brain and spinal cord in vivo. The modulations through glial cells often remain unnoticed since glial cells are mostly reduced or altered in these cell culture systems, even more so in primary hippocampal cell cultures containing in general fewer glial cells compared to spinal cord cultures. The sometimes contradictory findings obtained in apparently similar conditions raise the need for more standardized methods in association with multimodal approaches in basic experimental research and data interpretation leading to useful and valid conclusions and enhancing data reproducibility. Furthermore, the main majority of these experiments used rodent-derived cells or rodent models for their studies. Species-dependent structural and functional differences have to be taken into account when these results are translated to humans [75,76].

Herein it is also notable that several other studies point to further proteins present at the postsynaptic regions, such as scaffolding proteins and adhesion molecules, having the major impact on the induction of pre- and postsynaptic structures that were presented in great detail in a recent review, even proposing the molecular interactions of different classes of cell adhesion molecules as an independent mechanism of receptor and gephyrin cluster formation without any GABA or glycine release [8]. Moreover, super-resolution imaging techniques have shown that in addition to receptor clustering at the iPSD, the subsynaptic nanoscale organization of inhibitory receptors and gephyrin in SSDs opposite presynaptic transmitter release sites is also an important factor shaping synaptic strength and plasticity. Activity-dependent growth or shrinkage of inhibitory synapses might involve addition or removal, respectively, of these postsynaptic SSDs [10,58,59]. Whether these SSDs at inhibitory synapses are plastic also in vivo remains to be determined. Only the inclusion of all these components might allow in the future to develop the complete picture of GABAergic and glycinergic synapse formation.

## 9. Clinical Relevance

The activation of GlyRs and, to a certain degree, GABA_A_Rs by ligand binding generates molecular and cellular signals that contribute also to synaptogenesis and circuit computation. Dysfunctions within these steps due to various factors such as genetics, drug use, aging, and viral infections might have devastating effects on cellular communication, leading to network disruption within the central neural systems and muscle devastation in the periphery, underlying different pathologies. GlyR mutations resulting in dysfunction of the GlyRs are responsible for hyperekplexia, or startle disease, a neurological locomotion disorder, and are implicated in autism spectrum disorders. Some studies have also reported the role of glycine receptors in certain autoimmune diseases such as stiff person syndrome and high anxiety associated with Alzheimer’s disease [12]. It is important to note the value of studies carried out on mutated GlyRs to better understand GlyR dynamics at the synaptic level. Specifically, the work of Piro et al. shows how GlyR mutations cause structural changes in the interactions of GlyRβ with gephyrin, concluding that alterations in synaptic clustering of GlyR may potentially underlie the pathology of startle disease in patients that have such mutations in GlyRβ [77]. Further studies should clarify whether an altered ligand binding activation of mutated receptors is also part of the pathology. Recent advances in structural biology techniques have greatly enhanced understanding of the structural-functional properties of GyRs and elucidated novel allosteric binding sites as potential drug targets. Recently, several positive allosteric modulators of GlyRs were shown to have promising analgesic activity; however, their therapeutic application is impeded by difficulties in obtaining potent, selective modulators with favorable pharmacokinetic profiles and without side effects [78].

Ionotropic GABA_A_Rs are thought to be implicated in an even larger range of neurological disorders, including epilepsy, anxiety, depression, and sleep disorders, and to contribute to cognitive dysfunctions in Alzheimer’s disease, Parkinson’s disease, autism spectrum disorder, schizophrenia, etc. [79]. GABA_A_Rs can be positively or negatively modulated by different classes of endogenous and exogenous molecules, such as neurosteroids and benzodiazepines, showing therapeutic effects in some of these pathologic conditions. For example, the widely used benzodiazepines that target the α-γ subunit interface in the extracellular domain of the receptor [80] are attenuating seizures [81] and act as effective anxiolytics [82]. The potentiation of GABA-mediated currents by benzodiazepines and neurosteroids is caused by increased frequency of single-channel openings and duration of channel opening [80]. However, whether or not the potentiated ion currents of GABA_A_Rs might cause an increase in cluster size/number is not known; instead, a recent report has shown a time- and dose-dependent decrease in surface levels of GABA_A_Rs resulting in a loss of GABAergic synapses upon prolonged exposure to diazepam, one major benzodiazepine [83]. Since the pharmacological properties of GABA_A_Rs are determined by their subunit composition, due to the reduced subunit selectivity of presently available GABA_A_R-targeting drugs, they have several side effects, including dependence and withdrawal symptoms, and limited efficacy. Future studies have to decode the molecular mechanisms of subtype-specific inhibitory synapse formation and provide novel GABA_A_R pharmaceuticals to treat neurological disorders.

Dysfunctions related to gephyrin-mediated neurotransmission have been implicated in several neurological disorders, such as neurodevelopmental disorders, epilepsy, and even neurodegenerative diseases like Alzheimer’s [84,85,86,87,88]. Since extensive studies have been performed to recognize the pharmacological potential of inhibitory post-synaptic receptors, small molecules that target gephyrin were not identified until recently. Lately, the anti-malarial drug artemisinin and its semi-synthetic derivatives, collectively referred to as artemisinins, were discovered to directly interact with gephyrin and to modulate GABA_A_R signaling in pancreatic cells [89,90]. In our experiments artemisinins, when used in low doses, restored the number of postsynaptic gephyrin as well as GABA_A_R and GyR clusters in mouse models of cerebral amyloidosis [91,92]. Thus, these molecules could serve as potent gephyrin-specific modulators with therapeutic benefits in several neurological disorders [93].

In summary, deciphering molecular mechanisms that mediate the organization of pre- and postsynaptic specializations during inhibitory synapse formation, as well as understanding the relationships between GlyR and GABA_A_R deficits and neural system disorders, are essential for getting deeper insight into disease pathogenesis and for discovering new potential therapeutic targets, but also for improving existing therapeutics. In this context, a possible molecular mechanism linking the potentiation of GABA_A_R currents to alteration of receptor cluster sizes or even cluster number should be studied in more detail.

## 10. Conclusions

Reviewing the key publications from more than 25 years of research on the role of neurotransmitter-induced activity for the initiation and maintenance of glycine- and GABA_A_-receptor clustering at inhibitory synapses (Table 1), the following answers can be formulated to the initial questions that prompted this work:

1. The neurotransmitter-dependent activation of GlyRs seems to be essential for GlyR cluster formation, whereas several publications support that in the case of GABA_A_R cluster formation, the ligand-dependent activation is not essential for the initial postsynaptic clustering of these receptors (Figure 1 and Figure 2). Whether or not the capacity of GABA_A_Rs to form clusters independently from GABA binding is relevant under certain physiological conditions remains to be further elucidated.
2. It is very likely that under physiological conditions, the release of the neurotransmitter on both synapse types could regulate the postsynaptic receptor cluster formation: both transmitters are able to induce the formation of new GlyR or GABA_A_R clusters, and both transmitters seem to be sufficient to modulate the size of existing GlyRs or GABA_A_R clusters, respectively.
3. Glycine seems to be essential for the maintenance of existing GlyRs, whereas GABA seems to be dispensable for the maintenance of GABA_A_R clusters. Again, the interesting question of whether the independence of GABA_A_R clusters from continuous GABA activation is an important distinct feature of GABAergic synapses in comparison to GlyRs with relevancy in certain physiological conditions cannot be answered based on present knowledge.

Future experiments providing the missing elements are acutely needed to completely understand the cell biology of inhibitory synapse assembly, especially considering the pharmacological importance of both GlyRs and GABA_A_Rs [12,13].

## Figures and Tables

**Table 1:** Major publications presented in chronological order reporting the functional impact of the neurotransmitters glycine and GABA on clustering of the strychnine-sensitive GlyRs and GABA_A_Rs, respectively, as well as gephyrin in vitro and in vivo systems. Column 5 describes the most relevant results since in column 6 are cited original conclusions of the authors from the respective publications. Munc18-1: mammalian uncoordinated-18 protein; unc-25: *Caenorhabditis elegans* gene unc-25 encodes glutamic acid decarboxylase; hpf: hours post fertilization; dpf: days post-fertilization; vGAT: vesicular GABA transporter; GAD65: Glutamate decarboxylase 65 kDa isoform.

**Year**	**Authors/Title**	**System**	**Conditions**	**Results**	**Author’s Main Conclusions**	**Reference**
1998	Lévi S, Vannier C, and Triller A. Strychnine-sensitive stabilization of postsynaptic glycine receptor clusters	spinal cord neurons (rat) in vitro	Strychnine for 3, 7, 11 Div; 0.1, 1 and 10 uM Strychnine wash-out	GlyR number and size reduced, gephyrin clusters persisted at synapses	“…formation and/or stabilization of GlyR but not of gephyrin postsynaptic clusters depends upon functional GlyR.” “…the activation or conformational change of the GlyR is involved in the establishment and/or maintenance of the interaction between GlyR and gephyrin.”	[11]
1998	Kirsch J and Betz, H. Glycine-receptor activation is required for receptor clustering in spinal neurons	spinal cord neurons (rat) in vitro	Strychnine for 8 Div; 0.05, 0.1, and 0.5 μM block of L-type Ca^2+^ channels Strychnine wash-out	GlyR number reduced, gephyrin was removed from synapses	“…activation of GlyR is required for receptor clustering but not for the maintenance of differentiated postsynaptic sites.” “…gephyrin accumulation at glycinergic but not GABAergic synapses is prevented by strychnine.”	[12]
2000	Rao A, Cha EM, Craig AM. Mismatched appositions of presynaptic and postsynaptic components in isolated hippocampal neurons	hippocampal pyramidal cells (rat) deprived of GABA input in vitro (microisland cultures)	16–29 Div no GABAergic input	90% of pyramidal cells form GABA_A_ receptor clusters	“…GABAergic input is not necessary for the formation of GABAA receptor clusters.” “…both presynaptic and postsynaptic precursors can form independently of each other; i.e., neither component is necessary for formation of the other, but both can form spontaneously and later become aligned to form a functional synapse.”	[20]
2000	Verhage M, Maia AS, Plomp JJ, Brussaard AB, Heeroma JH, Vermeer H, Toonen RF, Hammer RE, van den Berg TK, Missler M, Geuze HJ, Südhof TC. Synaptic Assembly of the Brain in the Absence of Neurotransmitter Secretion	mice	deletion of Munc18-1, complete loss of neurotransmitter secretion	normal synapse formation, normal postsynaptic response in the absence of GABA, but degeneration without activation	“…synaptic connectivity does not depend on neurotransmitter secretion, but its maintenance does. Neurotransmitter secretion probably functions to validate already established synaptic connections.”	[21]
2002	Rasmussen H, Rasmussen T, Triller A, Vannier C. Strychnine-blocked glycine receptor is removed from synapses by a shift in insertion/degradation equilibrium.	spinal cord ventral horn neurons (rat) in vitro	short-term exposure of neurons to strychnine 10 μM	perinuclear accumulation of GlyRs within few hours, preceding the decrease in GlyR clusters at the cell surface	“…the normal turnover of the receptor is maintained in the presence of activity blockade because strychnine does not modify the rate of GlyR removal from postsynaptic sites via endocytosis.” “…activity may be required for gephyrin-dependent, synaptic anchoring of GlyR, it is not essential for the maintenance of this localization, once acquired.”	[13]
2003	Gally C, Bessereau JL. GABA is dispensable for the formation of junctional GABA receptor clusters in Caenorhabditis elegans	Caenorhabditis elegans GABAergic neuromuscular junctions	unc-25 mutants that do not synthesize GABA	GABA receptor clustering in mutant is identical to that in the wild type	“…at GABAergic neuromuscular junctions, GABA receptor clustering requires nerve–muscle interaction but not GABA neurotransmission.”	[22]
2005	Harms KJ, Craig AM Synapse composition and organization following chronic activity blockade in cultured hippocampal neurons.	hippocampal neurons in vitro (rat)	16–18 Div, chronic tetanus toxin to inhibit neurotransmitter release	postsynaptic recruitment of GABA_A_Rs, and gephyrin with normal density, size and distribution	“…activity and transmitter release are not necessary for the basics of glutamate or GABA synapse assembly.”	[23]
2009	Maas C, Belgardt D, Lee HK, Heisler FF, Lappe-Siefke C, Magiera MM, van Dijk J, Hausrat TJ, Janke C, and Kneussel M. Synaptic activation modifies microtubules underlying transport of postsynaptic cargo.	hippocampal neurons, (mouse and rat) mRFP-gephyrin	10–14 Div, Strychnine over 8 hs	mRFP-gephyrin signal numbers, sizes, and intensities reduced in neurites on GlyR blockade, mRFP-gephyrin accumulated in cell body clusters, impairment of neurite transport	“…synaptic activity regulates tubulin posttranslational modification, underlying intracellular transport of synaptic cargo, to determine the number of proteins available for synaptic transmission.”	[19]
2013	Yamanaka I, Miki M, Asakawa K, Kawakami K, Oda Y, Hirata H. Glycinergic transmission and postsynaptic activation of CaMKII are required for glycine receptor clustering in vivo.	zebrafish Mauthner cells	- Strychnine bath at 20~800 μM for various lengths of time during zebra-fish development: between 22 hpf and 31 dpf - nifedipine	GlyR number and density reduced, GlyR clusters are not maintained when strychnine is given at later stages when GlyR clusters have been already formed.	“…the formation and maintenance of GlyR clusters in the M-cells in the developing animals are regulated in a synaptic transmission-dependent manner, and CaMKII activation at the postsynapse is essential for GlyR clustering”.	[14]
2016	Oh WC, Lutzu S, Castillo PE, Kwon HB. De novo synaptogenesis induced by GABA in the developing mouse corte x	organotypic slice cultures from mouse somatosensory cortex acute cortical slices newborn mice	- two-photon GABA photolysis to mimic local GABA release in dendrites - GABA uncaging on layer 2/3 pyramidal neurons in vivo -GABA_A_R blockade by GABAzine - optogenetics	new gephyrin clusters formed and the amplitudes of uIPSCs subsequently increased reduced gephyrin clustering after blockade new gephyrin puncta and dendritic spines in young neurons	“…GABA is sufficient to drive inhibitory synapse formation. “…early-developing GABAergic inputs from cortical interneurons control both inhibitory and excitatory circuitry during cortical development.” “…early-depolarizing GABA action appears to promote local synaptogenesis and shapes cortical circuitry during brain development.”	[26]
2017	Nakahata Y, Eto K, Murakoshi H, Watanabe M, Kuriu T, Hirata H, Moorhouse AJ, Ishibashi H, Nabekura J. Activation-dependent rapid post-synaptic clustering of glycine receptors in mature spinal cord neurons	spinal cord neurons (mouse) in vitro	Strychnine over 14 Div; 1 μM Strychnine wash-out Local glycine (1 M) for 1 h	GlyR clusters with reduced size at inhibitory synapses return to control levels within 1 h of strychnine washout GlyR clustering at gephyrin-positive postsynapse	“In contrast to the current depolarization-dependent model of GlyR clustering, …the activation of GlyRs in more mature neurons…elicits changes in diffusion and increases in the postsynaptic GlyR clusters.” “…this phenomenon is dependent on PKC, but neither Ca^2+^ nor CaMKII activity.” “GlyR activation is more important for the formation of synaptic clustering of GlyR than for maintenance in mature neurons.”	[16]
2022	Burlingham SR, Wong NF, Peterkin L, Lubow L, Dos Santos Passos C, Benner O, Ghebrial M, Cast TP, Xu-Friedman MA, Südhof TC, Chanda S. Induction of synapse formation by de novo neurotransmitter synthesis	stem cells-derived human neurons in vivo mouse neurons of purely glutamatergic identity	ectopic expression of vGAT, GAD65, and GAD (V57 factors)	elevated the numbers of gephyrin clusters without changes in their sizes	“…presynaptic release of a neurotransmitter itself can signal the organization of relevant postsynaptic apparatus…”	[27]
2024	Carricaburu E, Benner O, Burlingham SR, Dos Santos Passos C, Hobaugh N, Karr CH, Chanda S. Gephyrin promotes autonomous assembly and synaptic localization of GABAergic postsynaptic components without presynaptic GABA release	Induced pluripotent stem cells (iPSC) reprogrammed into human glutamatergic neurons by a single transcription factor, Neurogenin-2	GABA-free cellular environment Gephyrin knock-out ectopic expression of V57 factors	postsynaptic GABA_A_R subunits and gephyrin clusters assembly elimination of GABA_A_Rs submembrane aggregation increased density of GAD-opposed gephyrin clusters	“…molecular organization of GABAergic postsynapses can initiate via a GABA-independent but Gephyrin-dependent intrinsic mechanism. “ “Gephyrin provides crucial structural support for postsynaptic assembly, regardless of GABA signals.” “Self-organizing GABAergic postsynaptic structures could be functionally stimulated by de novo biosynthesis and ectopic release of presynaptic GABA.”	[29]

## Abbreviations

AMPA	*α-Amino-3-hydroxy-5-methyl-4-isoxazolepropionic acid*
cAMP	cyclic adenosine monophosphate
CaMKII	Ca^2+^/calmodulin-dependent protein kinase type II
CDK5	cyclin-dependent kinase 5
Clptm1	cleft lip and palate transmembrane protein 1
3D-SIM	3D structured illumination microscope
dSTORM	direct Stochastic Optical Reconstruction Microscopy
EPSC	excitatory postsynaptic currents
GAD	glutamic acid decarboxylase
GABA	γ-aminobutyric acid
GABA_A_R	γ-aminobutyric acid A-receptors
GARLH	GABA_A_R regulatory LHPFL family protein
GyR	glycine receptor
GSK3β	glycogen Synthase Kinase 3 beta
HFU	High-frequency uncaging
iPSD	inhibitory postsynaptic density
IPSC	inhibitory postsynaptic currents
LHFPL4	lipoma HMGIC fusion partner-like 4
Munc18-1	mammal *unc*-18-1 (Munc18-1 or n-Sec1; herein M18)
NLG2	neuroligin 2
NMDA	N-methyl D-aspartate
PKA	protein kinase A
PKC	protein kinase C
RER	rough endoplasmic reticulum
SIM	Structured Illumination Microscopy
Shisa-7	Shisa family member 7
SNARE	soluble N-ethylmaleimide-sensitive factor attachment protein receptor
SSD	subsynaptic domain
uIPSCs	uncaging-evoked inhibitory postsynaptic currents
VIAAT	Vesicular inhibitory amino acid transporter
VAMP2	Vesicle-associated membrane protein 2
